# Comparison of mathematically arterialised venous blood gas sampling with arterial, capillary, and venous sampling in adult patients with hypercapnic respiratory failure: a single-centre longitudinal cohort study

**DOI:** 10.1136/bmjresp-2022-001537

**Published:** 2023-06-27

**Authors:** Michael Gordon Davies, Dariusz Rafal Wozniak, Timothy George Quinnell, Earl Palas, Susan George, Yingchang Huang, Ruwanthi Jayasekara, Victoria Stoneman, Ian Edward Smith, Lars Pilegaard Thomsen, Stephen Edward Rees

**Affiliations:** 1 Respiratory Support and Sleep Centre, Royal Papworth Hospital NHS Foundation Trust, Cambridge, UK; 2 Respiratory and Critical Care group, Aalborg University, Aalborg, Denmark

**Keywords:** Non invasive ventilation, Respiratory Measurement, Assisted Ventilation

## Abstract

**Background:**

Accurate arterial blood gas (ABG) analysis is essential in the management of patients with hypercapnic respiratory failure, but repeated sampling requires technical expertise and is painful. Missed sampling is common and has a negative impact on patient care. A newer venous to arterial conversion method (v-TAC, Roche) uses mathematical models of acid–base chemistry, a venous blood gas sample and peripheral blood oxygen saturation to calculate arterial acid–base status. It has the potential to replace routine ABG sampling for selected patient cohorts. The aim of this study was to compare v-TAC with ABG, capillary and venous sampling in a patient cohort referred to start non-invasive ventilation (NIV).

**Methods:**

Recruited patients underwent near simultaneous ABG, capillary blood gas (CBG) and venous blood gas (VBG) sampling at day 0, and up to two further occasions (day 1 NIV and discharge). The primary outcome was the reliability of v-TAC sampling compared with ABG, via Bland-Altman analysis, to identify respiratory failure (via PaCO_2_) and to detect changes in PaCO_2_ in response to NIV. Secondary outcomes included agreements with pH, sampling success rates and pain.

**Results:**

The agreement between ABG and v-TAC/venous PaCO_2_ was assessed for 119 matched sampling episodes and 105 between ABG and CBG. Close agreement was shown for v-TAC (mean difference (SD) 0.01 (0.5) kPa), but not for CBG (−0.75 (0.69) kPa) or VBG (+1.00 (0.90) kPa). Longitudinal data for 32 patients started on NIV showed the closest agreement for ABG and v-TAC (R^2^=0.61). v-TAC sampling had the highest first-time success rate (88%) and was less painful than arterial (p<0.0001).

**Conclusion:**

Mathematical arterialisation of venous samples was easier to obtain and less painful than ABG sampling. Results showed close agreement for PaCO2 and pH and tracked well longitudinally such that the v-TAC method could replace routine ABG testing to recognise and monitor patients with hypercapnic respiratory failure.

**Trial registration number:**

NCT04072848; www.clinicaltrials.gov

**WHAT IS ALREADY KNOWN ON THIS TOPIC**

- Individuals admitted to hospital with acute respiratory problems often require arterial blood gas (ABG) sampling, though national audits have shown important deficiencies in care associated with omission or delay in ABG testing.
- Prior studies have shown that a model for mathematically converted peripheral venous samples (v-TAC) shows close agreement to arterial values, though its potential role is uncertain. This study explores the role of v-TAC in the assessment and monitoring of patients with respiratory failure who were treated with non-invasive ventilation.

**WHAT THIS STUDY ADDS**

- v-TAC calculated sampling of PaCO_2_ agreed well with arterial measurements for both recognising hypercapnic respiratory failure and monitoring the response to non-invasive ventilation. In contrast, capillary and venous blood gas sampling performed less well against ABG.
- Sampling via v-TAC was less painful and had a higher first-time success rate compared with ABG.

**HOW THIS STUDY MIGHT AFFECT RESEARCH, PRACTICE OR POLICY**

- Integration of the v-TAC method into routine clinical practice could simplify and improve the assessment and management of individuals with suspected acute hypercapnic respiratory failure, a major reason for acute hospitalisation.

## Introduction

Accurate arterial blood gas (ABG) analysis is essential in the management of hypercapnic respiratory failure,^1^ but sampling is painful,^2^ depending on operator expertise. Patients often require multiple samples over time to assess therapy response. Use of local anaesthetic is rare in UK ward-based practice despite longstanding recommendations for its use in national guidance.^3^ This recommendation was reinforced in the British Thoracic Society’s 2017 guideline for oxygen use in adults in healthcare and emergency settings, which recommended (grade A) use of ‘local anaesthesia for all ABG specimens except in emergencies’.^4^

Arterialised capillary blood gas (CBG) samples present an alternative to this accepted standard, though their reliability and accuracy are uncertain. Venous blood gas (VBG) pH sampling can screen acutely unwell patients,^5^ but has less precision for PCO_2_. VBG testing does not replace subsequent ABG sampling if treatment with acute non-invasive ventilation (NIV) is considered.

A newer venous to arterial conversion method (v-TAC, Roche) is available for clinical use.^6^ It calculates arterial acid–base status from VBG measurements combined with blood oxygen saturation (SpO_2_) from standard pulse oximetry using mathematical models of acid–base chemistry.^7^ Peripheral venous blood is mathematically transported back through the tissues adding oxygen and removing carbon dioxide in a fixed ratio until oxygen levels match measurements taken from the pulse oximeter.^6^ A mathematical model of acid–base is applied to perform this calculation, which accounts for the oxygen dissociation curve, calibrating this to the venous blood, and for the Bohr-Haldane effects in the blood.^7^ The model calculates ‘arterialised’ values of pH, PCO_2_, PO_2_ and SO_2_ assuming a respiratory quotient of 0.82, and adding oxygen and removing carbon dioxide until the calculated SO_2_ is equivalent to measured SpO_2_. The method essentially aims to compensate for the effects of aerobic metabolism. It is important to note that v-TAC calculations of arterial PO_2_ are inaccurate when the plateau of the oxygen dissociation curve is reached, typically when SpO_2_ is greater than 97%.

Prior studies confirm that v-TAC shows close agreement with ABG for pH and PCO_2_ at all ranges,^8–15^ though its clinical application is not established. The v-TAC method also shows close agreement with ABG for PO_2_ up to 10 kPa, though not for higher oxygen levels. This is because of the nature of the oxygen dissociation curve. The commercial version of the software, therefore, reports PO_2_ up to 10 kPa. The primary aim of our study was to compare ABG sampling against VBG, v-TAC and CBG sampling in the longitudinal assessment of inpatients with suspected hypercapnic respiratory failure and considered for treatment with NIV.

While ABG sampling is the recognised gold standard, our study data also provided a means to explore this further as a secondary aim. There is emerging evidence that intermittent ABG sampling can lack precision due to transient changes in the breathing pattern of the patient. A recent study of invasively ventilated adult subjects showed that arterial blood pH and CO_2_ values change rapidly in response to a transient increase (+100%) or decrease (−60%) in ventilation, whereas peripheral venous samples change little in the first 60 s.^16^ It is well recognised that the anticipatory fear of pain just before an ABG can cause hyperventilation or breath-holding.^17 18^ Data are lacking on the prevalence or impact. It was postulated recently that the v-TAC conversion from venous blood could mitigate the acute changes in the arterial baseline induced by a transient change in breathing pattern.^19^ We, therefore, conducted a post hoc analysis of our data set to consider the potential effect of transient changes in breathing pattern.

## Methods

### Participants

From January 2019 to October 2020, we screened patients aged 18+ years who were admitted on an inpatient basis for home NIV assessment and due to undergo ABG sampling as part of their routine care. The exclusion criteria were as follows: (1) inability to provide informed consent, (2) clinical necessity to perform blood gas sampling prior to allowing sufficient time for informed consent and (3) inability to obtain reliable SpO_2_ readings.

All measurements were undertaken during a single admission episode. Recruited patients underwent near simultaneous ABG, CBG and VBG sampling at day 0, and up to two further occasions (day 1 NIV and discharge) if NIV was indicated. Pulse oximetry was recorded at the exact point of sampling.

Participant experience of each type of blood sampling method was recorded using a standard visual/analogue pain score (scale 0–10).

### Patient and public involvement

We did not include formal patient and public involvement in study design.

### Sample handling

Staff taking the sampling were suitably qualified according to hospital protocol and experience. ABG and VBG samples were typically undertaken by doctors and advanced nurse practitioners. Local anaesthesia was not used routinely, although was permitted according to clinician/patient preference. CBG samples were typically obtained by registered nurses and healthcare assistants via the earlobe and following the application of a vasodilator cream to each earlobe (Deep Heat Cream, Atco, UK) until it was red and warm (up to 20 min before blood sampling). Pulse oximetry was performed initially in both hands to confirm uniformity. It was then performed continuously during the procedure on the non-VBG arm. A tourniquet was used for venous sampling, though not for prolonged periods. SpO_2_ was recorded for the exact time that the sample was achieved. Blood sampling was as simultaneous as possible.

Samples were processed immediately via blood gas analyser (Radiometer ABL90 Flex, Radiometer Medical, Denmark). The venous to arterial conversion was performed using v-TAC software (OBI Medical, Denmark) following the subject’s hospital admission.

Samples that were rejected by the analyser (insufficient volume for example) were recorded as a failed sample. In addition, the following prespecified criteria were used to exclude samples affected by obvious preanalytical error: (1) a lower (4% or more) arterial value for SaO_2_ via co-oximetry compared with the contemporaneous peripheral saturation (SpO_2_), indicating venous contamination during the arterial draw,^20 21^ (2) a difference in haemoglobin concentration between arterial and venous samples of 25% or more and/or an absolute difference greater than 1.5 mmol/L, indicating excessive sample sedimentation in either the venous or arterial sample and (3) in the event of no pulse oximetry, which means that the v-TAC conversion is not possible.

### Outcomes

The primary outcome was the agreement of v-TAC sampling compared with ABG with respect to the changes in PaCO_2_ in response to NIV.

### Statistical analysis

Prior studies exploring the agreement between absolute arterial and v-TAC PaCO_2_ values indicated a requirement for 41 matched samples at 80% power (β-level 0.20) and 5% significance (α-level 0.05).^8–15^ We assumed a proportionate variability for the change in PaCO_2_ in the absence of existing data, though aimed to recruit at least 80 patients. In addition to the lack of data, this was because some recruited patients would not require NIV; a review of admissions to our unit in the preceding year showed that 67% of patients assessed were treated with NIV. We elected to include patients prior to NIV because (1) this enabled the agreement analysis to include a wider range of baseline CO_2_ values, (2) we wished to minimise sampling burden for patients and (3) all matched sample episodes meeting prespecified technical criteria would be available for the agreement analysis for ABG vs v-TAC, CBG and VBG. Bland-Altman analysis of mean and limits of agreement, and analysis of response to NIV, were performed for time-matched samples for values of pH, PaCO_2_ and PaO_2_. Pain scores were compared via Friedman’s two-way analysis of variance by ranks (IBM SPSS Statistics for Windows, V.27) and significant values adjusted for by the Bonferroni correction.

## Results

**Figure 1:**
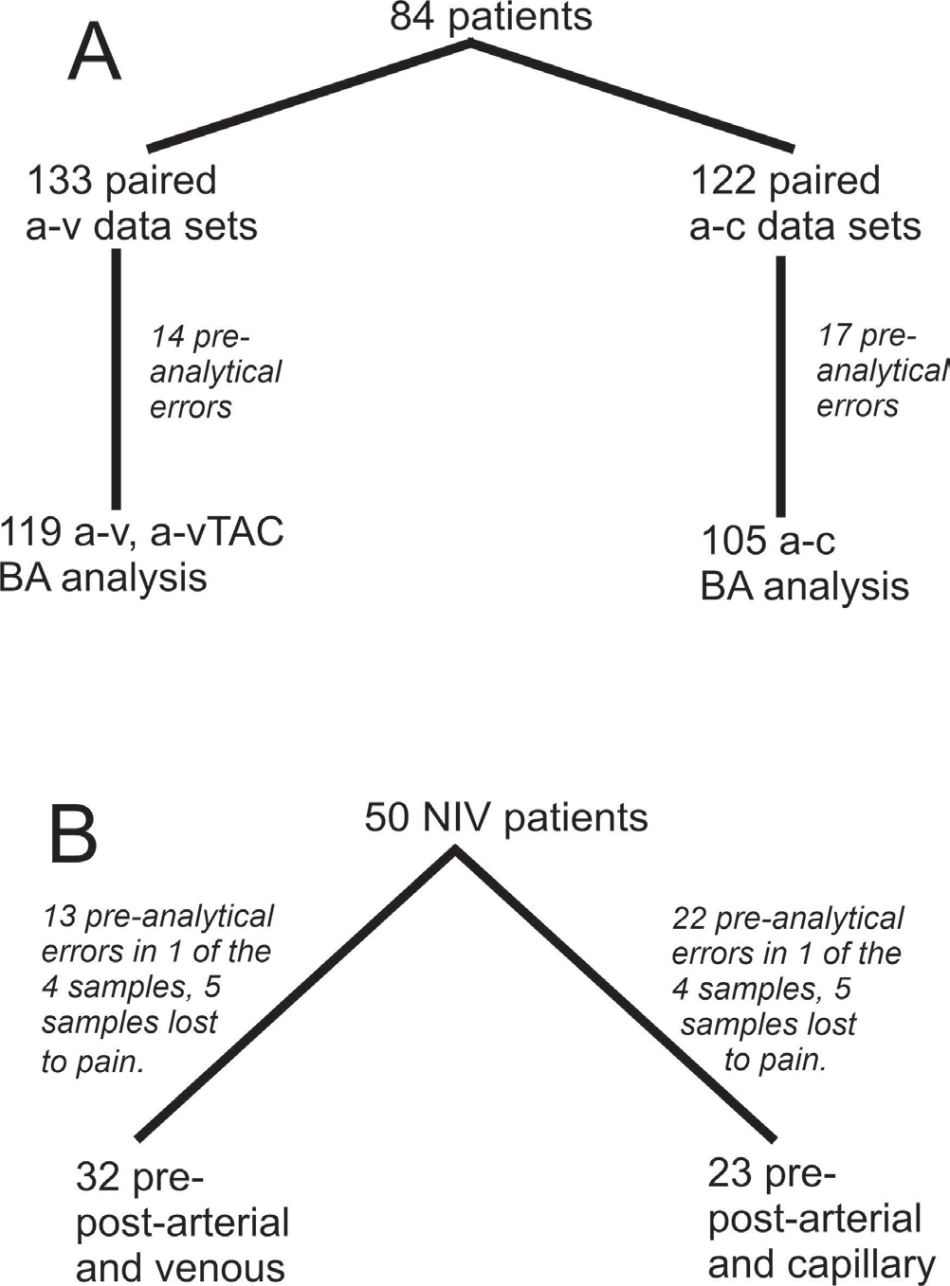
Consort diagram to show patient recruitment and data availability for analyses. Part A shows total recruitment and the number of matched samples available for Bland-Altmann agreement analysis (a-v=arterial-venous, a-v-TAC = arterial-v-TAC, a-c=arterial-capillary). In total, 50 patients started NIV. Successful pre-NIV and post-NIV sampling was available for 32 subjects for the arterial—venous/v-TAC comparison and 23 for the arterial-capillary comparison. NIV, non-invasive ventilation; v-TAC, venous to arterial conversion.

Figure 1 shows trial recruitment. We recruited 84 patients (45% women), of whom 50 started NIV. Primary diagnoses for respiratory failure were Chronic Obstructive Pulmonary Disease (50%), obesity (26%), neuromuscular (17%) and others (7%).

Sampling was attempted on 216 (ABG), 179 (VBG) and 215 (CBG) occasions and successful on 138, 149 and 122 occasions, respectively. Sampling success was lowest for CBG and highest for VBG (table 1). Patients found consecutive sampling easier to tolerate. This introduced some variance in sample timing, with an average (mean and median) of 6 min between ABG and VBG. Pain scores were significantly higher for ABG compared with VBG (p<0.0001) and CBG (p<0.0001). VBG was more painful than CBG (p=0.012).

**Table 1:** Sampling attempts and pain scores

	ABG	VBG (v-TAC)	CBG
Sample success rates			
Success at first attempt (%)	67	88	55
Success on second attempt (%)	15	8	19
Success on three or more attempts (%)	5	2	5
Failure to obtain sample or patient refusal (%)	13	2	21
Average number of practitioner attempts to achieve a successful sample (n)	1.56	1.18	1.81
Pain from sampling			
Mean Pain Score (SD)	4.3 (3)	2.4 (2.2)	1.3 (1.6)

ABG, arterial blood gas; CBG, capillary blood gas; VBG, venous blood gas; v-TAC, venous to arterial conversion.

For the Bland-Altmann analysis of agreement, paired ABG and VBG (v-TAC) sampling was achieved for 133 samples, of which 119 (89%) passed prespecified technical criteria for trueness of sample. Of the 14 exclusions, 8 were because of venous contamination of the ABG, 5 were because of excessive sedimentation and 1 was excluded because of no simultaneous pulse oximetry value. Values excluded due to venous contamination are shown in table 2.

**Table 2:** Details of ABG samples excluded due to venous contamination as indicated by large differences between SaO_2_ and SpO_2_

Excluded sample	SaO_2_(%) via co-oximetry (blood gas analyser)	PaO_2_ (kPa) (ABG result)	SpO_2_ (%) via peripheral pulse oximetry (sats metre)	PaO_2_ (kPa) (v-TAC conversion of venous sample)
1	59.2	4.36	86	7.29
2	65.9	5.22	93	8.13
3	60.8	4.89	93	9.19
4	85.7	6.82	94	9.44
5	83	6.04	92	9.30
6	84.2	6.97	92	8.79
7	85.3	6.95	90	8.17
8	85.8	6.91	91	7.83

ABG, arterial blood gas; v-TAC, venous to arterial conversion.

**Figure 2:**
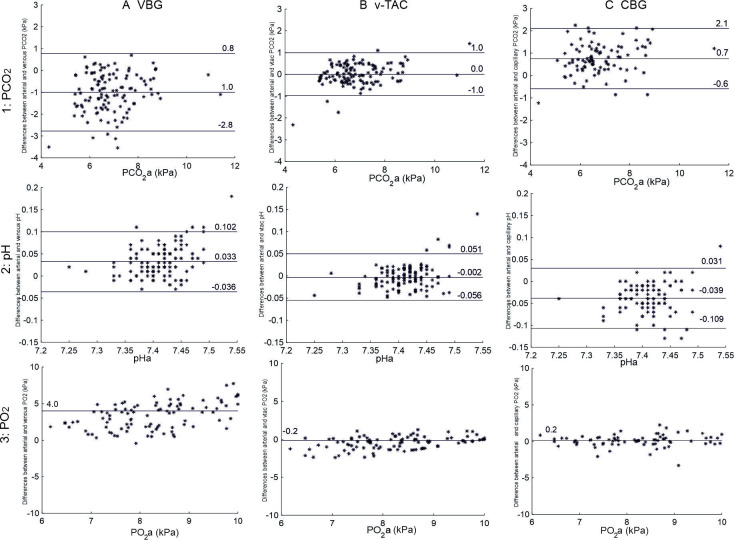
Bland-Altmann agreements between ABG sampling and VBG (column A), v-TAC (column B), and CBG (column C) with respect to PCO_2_ (row 1), pH (row 2), and PO_2_ (row 3). Mean differences and limits of agreement are shown for PCO_2_ and pH. PO_2_ values were not normally distributed, so limits of agreement are not displayed. Note that v-TAC does not convert PO_2_ values >10 kPa, hence PO_2_ data are shown to 10 kPa. ABG, arterial blood gas; CBG, capillary blood gas; VBG, venous blood gas; v-TAC, venous to arterial conversion.

Figure 2 shows Bland-Altman agreements for ABG against VBG, v-TAC and CBG and compares changes in PCO_2_ pre-NIV and post-NIV. ABG and v-TAC values showed close agreement for PCO_2_ and pH values. Mean bias for v-TAC against ABG was 0.01 kPa (SD 0.50 kPa), whereas it was −1.00 (0.90) kPa for VBG and +0.75 (0.69) kPa for CBG. For pH, the bias vs ABG was −0.002 (0.027) for v-TAC, 0.033 (0.035) for VBG and −0.039 (0.035) for CBG (a negative value for mean difference indicates that the arterial value is lower than the comparator).

**Figure 3:**
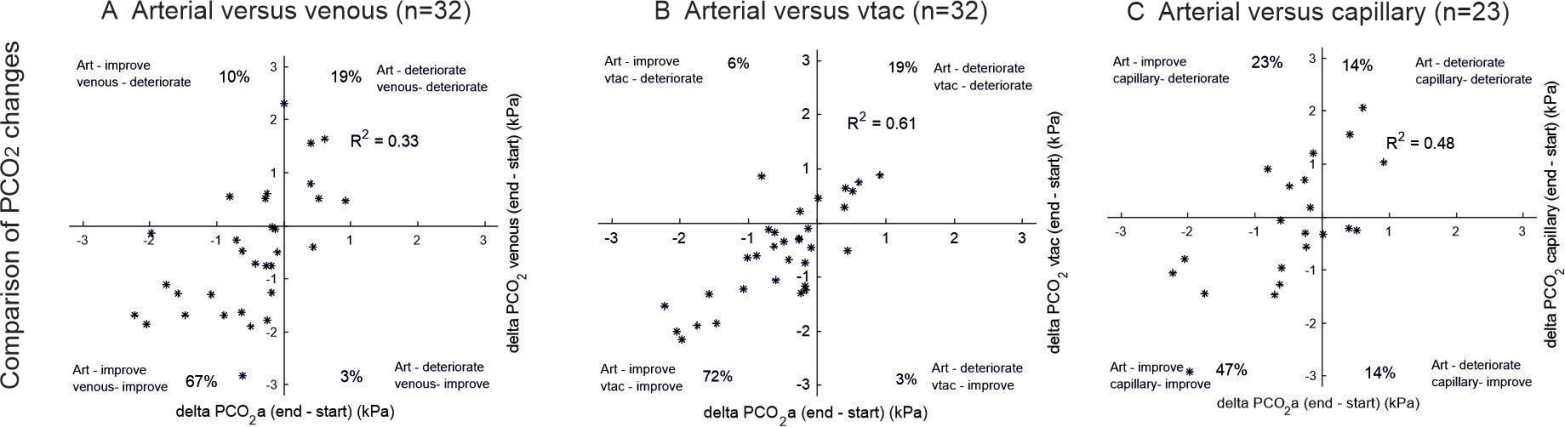
Arterial PCO_2_ changes due to NIV compared with the changes seen for VBG (column A), v-TAC (column B), and CBG (column C). Categorical agreement (for example, a reduction in PCO_2_ in both comparators) was 86% for VBG, 91% for v-TAC, and 61% for CBG. R^2^ values were highest for v-TAC (0.61). CBG, capillary blood gas; VBG, venous blood gas; v-TAC, venous to arterial conversion.

The CO_2_ response to ventilation according to sampling method was assessed for patients who started NIV and achieved successful pre and post NIV sampling. Data were available for 32 patients for the arterial and venous/v-TAC comparison, and for 23 for the arterial and capillary comparison. Figure 3 shows that arterial sampling was most closely associated with v-TAC with respect to absolute pre and post NIV values and the change in PaCO_2_. Compared with CBG and VBG, v-TAC classified the post-NIV improvement or deterioration in PCO_2_ most accurately (91%, R^2^=0.61). In addition, we assessed the mean change in CO_2_ in response to NIV according to sampling method; it was 0.53 (SD 0.81) kPa for ABG, 0.55 (0.87) kPa for v-TAC, 0.49 (1.20) kPa for VBG and 0.16 (1.18) kPa for CBG.

We then considered the potential impact of unstable ventilation around the time of ABG sampling. We defined relative hyperventilation and hypoventilation as follows:

Hyperventilation: pHa-pHv≥0.09 and PCO2a-PCO2v≤−1.5 kPa.

Hypoventilation: pHa-pHv≤−0.01 and PCO2a – PCO2v≥0.1 kPa.

Using these criteria, we found evidence of hyperventilation for 8/119 paired samples, and hypoventilation for 10/119 paired samples (ie, total affected=18/119 (15%) samples).

## Discussion

ABG sampling is a common test for patients admitted to hospital with acute respiratory problems. National audits, however, have repeatedly shown important deficiencies in care associated with delayed or missed blood gas sampling.^22 23^ A National Confidential Enquiry into Patient Outcome and Death study showed that there was a delay in starting NIV for 27% of patients assessed. Failure to recognise respiratory failure due to a lack of blood gas testing was the most common reason for treatment delay.^24^

In this study, patients with known or suspected hypercapnic respiratory failure underwent serial blood gas monitoring during treatment with NIV. We assessed v-TAC, a novel method of mathematically arterialised venous blood gas sampling, against arterial, capillary and venous blood gas sampling. We found that the v-TAC method showed close agreement with arterial PaCO_2_ and pH results, the key physiological variables used to guide treatment with NIV. In addition, the v-TAC method showed good agreement with arterial for PO_2_ values up to 10 kPa. In contrast, average bias in PCO_2_ was −0.75 kPa for capillary sampling (reading lower than arterial) and +1 kPa for VBG sampling (reading higher than arterial). CIs were wider for CBG and VBG compared with v-TAC, further reducing their reliability as surrogates for arterial sampling.

An important aspect of this study was to follow patients longitudinally after starting treatment with NIV. While guidelines recommend repeated ABG sampling to assess NIV response, CBG or VBG is often used in clinical practice because of their greater convenience. We found that v-TAC tracked well with ABG sampling, whereas VBG and CBG sampling did not, introducing a bias and wider variation compared with ABG. CBG measurements functioned worst of all, with only 61% of samples matching the same categorical change in ABG in response to NIV.

We also found that arterial sampling was the most painful with its median score (4) reaching the moderate pain category. If considered alongside the relatively low first-time success rate (67%), this reinforces guideline recommendations to use local anaesthesia.^3 4^ Capillary sampling, while not painful in comparison, showed lower sampling overall success rates.

It is important to acknowledge several limitations. First, the v-TAC calculation is based on a compensation for normal aerobic metabolism. The method assumes peripheral venous sampling is taken from a limb with a clearly recognisable pulse and a normal capillary response. It would not be suitable in clinical scenarios characterised by additional anaerobic metabolism at the tissue site. In the setting of haemodynamic instability or sepsis, for example, then arterial sampling is preferred. We also emphasise here that calculation of higher values of PO_2_ via v-TAC is not as robust as for pH and PCO_2_. For other patient cohorts, where O_2_ evaluation is critical, then an arterial sample remains most appropriate.

Second, the single-centre nature of our study limits its generalisability. There are a growing number of single-centre studies that show successful application of the v-TAC method. These have served to establish the safety and reliability of the method and to refine the patient groups most likely to benefit. Ultimately, it could replace most, but not all, arterial samples for some patient groups. This would be quite a significant change in the patient pathway given that arterial sampling is the current gold standard. We believe that the available data support the introduction of v-TAC-directed pathways into clinical care, though would recommend that implementation should be assessed via service evaluation or further study.

One of the issues of comparing against a gold standard is in assuring the gold standard itself. Arterial sampling is subjected to known errors, and it would not be sensible to compare against a non-physiological sample. We considered significant venous admixture and sedimentation to be the two main preanalytical errors that might affect an ABG result. We used conservative criteria to exclude clear examples of preanalytical error, without excluding those with more equivocal changes. Using our criteria, we excluded 8/133 (6%) of presumed arterial samples on the grounds of venous contamination.

There is also inherent variability of sampling even when the sample is clearly arterial.^15 25^ As a pragmatic study, we accepted that patients were unlikely to be physiologically stable from a respiratory perspective at the time of sampling. We did not tend to use local anaesthesia for ABG sampling and recognise this as a weakness against national standards^3 4^ though not in comparison to usual practice in the UK. Transient hyperventilation or breath-holding around the time of blood sampling is common^17 18^ and may affect ABG results^16^; our post hoc analysis suggests that this may have been the case for up to 15% of ABG samples obtained.

## Conclusions

For a cohort of patients treated with NIV for hypercapnic respiratory failure, a method of mathematical arterialisation of a venous sample (v-TAC) was interchangeable with arterial sampling for PCO_2_ and pH assessment. The v-TAC method was less painful than ABG and had a higher likelihood of sampling success. The use of capillary or venous blood gas testing was found to be inaccurate and is not recommended as surrogates for ABG measurement. Our data also suggest that up to 15% of ABG sample results may be affected by a transient change in breathing pattern, reinforcing the use of local anaesthesia if intermittent ABG sampling is undertaken. Further studies under more controlled conditions are needed to explore the true variability of arterial results. The v-TAC method offers significant promise within acute clinical pathways for respiratory patients where its implementation could simplify the patient pathway and improve the recognition and monitoring of respiratory failure.

## Data Availability

All data relevant to the study are included in the article or uploaded as supplementary information.
